# Orbital mucormycosis in immunocompetent children; review of risk factors, diagnosis, and treatment approach

**DOI:** 10.1186/s12879-020-05460-2

**Published:** 2020-10-19

**Authors:** Ali Amanati, Hamide Barzegar, Gholamreza Pouladfar, Anahita Sanaei Dashti, Mohamad Bagher Abtahi, Bijan Khademi, Mohammad Javad Ashraf, Parisa Badiee, Seyedeh Sedigheh Hamzavi, Ali Kashkooe

**Affiliations:** 1 Professor Alborzi Clinical Microbiology Research Center, Shiraz University of Medical Sciences, Shiraz, Iran; 2 Shiraz University of Medical Sciences, Namazi Hospital, 7193711351, Zand Ave, Shiraz, Iran; 3 Department and Research Center of Otolaryngology, Head and Neck Surgery, Shiraz University of Medical Sciences, Shiraz, Iran; 4 Department of Pathology, Shiraz University of Medical Sciences, Shiraz, Iran

**Keywords:** Mucormycosis, Orbital, Immunocompetent, Children

## Abstract

**Background:**

Orbital mucormycosis is a rare but potentially severe and troublesome invasive fungal infection that could be occurred even in healthy individuals. The initial clinical presentation is similar to bacterial pre-septal or septal cellulitis, especially in early stages.

**Case presentation:**

Herein, we describe the successful management of a series of five cases presenting with orbital mucormycosis in previously healthy children.

**Conclusions:**

Orbital mucormycosis is extremely rare in healthy children and maybe life-threatening when diagnosis delayed given a similar clinical presentation with bacterial septal cellulitis. Intravenous antifungal therapy with amphotericin B and timely surgical drainage is live-saving.

## Introduction

Mucormycosis is the third most common invasive fungal infections following candidiasis and aspergillosis [1]. Mucormycosis is a life-threatening infection that could manifest as a local or systemic infection. Due to high mortality and morbidity, early diagnosis and treatment are crucial. One of the most common forms of mucormycosis is rhino-orbito-cerebral infection [2, 3]. Local pain, chemosis, multiple cranial nerve palsies, unilateral peri-orbital facial pain, blepharoptosis, proptosis, acute ocular motility changes, ophthalmoplegia, headache, and acute vision loss are the most common signs and symptoms [1]. Diagnosis usually based on the identification of organisms in tissue by histopathology, culture, KOH preparation and molecular tests [4]. Surgical debridement, in addition to systemic antifungal therapy, is the standard treatment approach which should be started early after diagnosis [5, 6]. In this case series, we present five previously healthy children, primarily presented with orbital cellulitis. Successful treatment and complete cure occurred despite a late diagnosis in all cases.

## Case presentation

### Case 1

The 7-month-old girl visited with edema and erythema in the left inferomedial canthus for 6 weeks before admission. Her mother told that they were villagers and lived in a rural place near Shiraz city. They were on vacation in an area around their home. In the spiral orbital computed tomography (CT) scan with contrast, peri-orbital abscess formation, and evidence of dacryocystitis found. She scheduled promptly for orbital biopsy and abscess drainage. The results of the biopsy and polymerase chain reaction (PCR) was positive for mucormycosis. Accordingly, systemic amphotericin-B deoxycholate started. Surgical debridement repeated a few days later, given the inadequate clinical response. She discharged after 2-month hospitalization.

### Case 2

A 9-month-year-old girl visited with swelling of the right inferomedial canthus for 6-week before hospital admission. The patient had a history of repeated episodes of epiphora, and she was suspicious of nasolacrimal duct obstruction (NLDO). She had a history of probing and 17-day hospitalization in another university-affiliated hospital. One week before admission, her condition got worsen, and she developed peri-orbital edema, and erythema extended to the superior side of the right medial canthus. Marcus Gunn (a relative afferent pupillary defect (Marcus Gunn pupil) which indicates an asymmetric, prechiasmatic, afferent conduction defect [7]) was 1(+), and severe peri-orbital edema with severe proptosis detected without any extra-ocular muscle (EOM) disturbance. Mucormycosis cultured on culture and confirmed by histopathologic examination.

### Case 3

18-month boy with right eye proptosis, purulent discharge, and peri-orbital swelling and redness visited in Nemazee hospital. His mother told that his first symptoms begin since 1-month before admission. Mucormycosis confirmed by histopathologic examination and culture. He went under debulking five times during his hospitalization in addition to systemic antifungal therapy.

### Case 4

A 10-month boy was visited by a general physician due to high-grade fever and managed primarily as the common cold. His mother noticed the right peri-orbital erythema after 2 days. Ipsilateral peri-orbital swelling developed gradually. Peri-orbital abscess formation diagnosed on CT-scan, and he underwent emergency debridement. Histopathologic examination revealed non-septate broad hyphae and tissue PCR become positive for mucormycosis.

### Case 5

An ophthalmologist referred to a 3-year-old previously healthy boy with peri-orbital swelling to Nemazee hospital. He developed acute onset left sub-orbital swelling without significant pain and fever since 6 weeks ago. The child had a history of frequent exposure to corn and fodder storage and hay. Spiral orbital CT-scan performed, which revealed an orbital abscess in the medial aspect of left orbit without any bony destruction or concomitant sinusitis. The patient scheduled for anterior orbitotomy and incisional biopsy. Despite effective antifungal treatment, the patient needed subsequent surgical debridement.

Demographics, duration of symptoms before admission, clinical features, comorbidities, diagnostic approach, predisposing factors, treatment strategy, follow-up and outcome of studied pediatric patients and also the available previous reports on orbital mucormycosis in healthy individuals are summarized in Table 1.

**Table 1:** Summary characteristics of patients with isolated orbital Mucormycosis

	Year^**a**^	Age	Sex	The onset of symptoms PTA	Clinical features	Ophthal. exam	Concurrent sinusitis	Diagnostic approach ^**1**^	Predisposing factors	Treatment strategy	Treatment duration	Surgical debridement	Follow-up and outcome
**Our case series**													
**1**	2017	7/M	F	6/W	Dacryocystitis + Periorbital cellulitis	Left conjunctival injection and firm fixed mass on the inferomedial aspect of the left canthus	Yes	Pathology, fungal culture, KOH, molecular confirmation	Gardening	DAmB (1.5 mg/kg/day)/ LAmB (5 mg/kg/day)	8/W	Orbitotomy+ debulking (three times)	3/Y, Complete cure
**2**	2017	9/M	F	1/W	Dacryocystitis + Periorbital cellulitis	Peri-orbital edema, and erythema extended to the superior side of the right medial canthus	Yes	Pathology, fungal culture, KOH, molecular confirmation	Trauma (nasolacrimal duct probing)	DAmB (1.5 mg/kg/day)/ LAmB (5 mg/kg/day)	9/W	Orbitotomy+ debulking (two times)	3/Y, Complete cure
**3**	2018	18/M	M	4/W	Orbital cellulitis	Right eye proptosis, purulent discharge, and peri-orbital swelling and redness	Yes	Pathology, fungal culture, KOH, molecular confirmation	Not identified	LAmB (5 mg/kg/day)/ PSC ^2^ (200 mg/q 6 h)	17/W	Orbitotomy+ debulking (five times)	2/Y, Complete cure
**4**	2019	10/M	M	1/W	Periorbital cellulitis	Right peri-orbital erythema and swelling	No	Pathology, fungal culture, KOH, molecular confirmation	Not identified	DAmB (1.5 mg/kg/day)/ LAmB (5 mg/kg/day)/PSC (200 mg/q 6 h)	12/W	Orbitotomy+ debulking	9/M, Complete cure
**5**	2019	3/Y	M	6/W	Periorbital cellulitis	Left sub-orbital swelling	No	Pathology, fungal culture, KOH, molecular confirmation	Frequent exposure to corn and fodder storage and hay	DAmB (1.5 mg/kg/day)/ LAmB (5 mg/kg/day)/ PSC (200 mg/q 6 h)	12/W	Orbitotomy+ debulking (four times)	5/M, Complete cure
**Previous case reports**													
**6**	2012 [8]	2/Y	M	18/days	Periorbital cellulitis		NA	Pathology, fungal culture, KOH	Not reported	DAmB, ICZ	6/M	Anterior orbitotomy and debulking	2/Y, Complete cure
**7**	1951 [9]	10/Y	M	NA	Exudative retinitis		NA	Pathology	Coat’s disease	NA	NA	Enucleation	NA
**8**	2009 [10]	71	M	1/day	Orbital cellulitis		NA	Pathology	Diabetes mellitus, non-Hodgkin lymphoma of diffuse large B-cell (DLCBL)	LAmB, PSC	About 40/days	Extensive surgical debridement	Death
**9**	2014 [11]	13	M	3/W	Periorbital cellulitis		NA	Pathology	Gardening, immunocompetent, concurrent corticosteroid therapy for approximately 1/M	DAmB	26/days	Orbital exenteration	Cure with morbidity

*Abbreviations*: *M* male, *F* female, *PTA* prior to admission, *Ophthal. exam* ophthalmic examination, *DAmB* deoxycholate amphotericin B, *LAmB* liposomal amphotericin B, *PSC* posaconazole, *ICZ* Itraconazole;

^a^Presentation year [reference]

^1^Including standard diagnostic tests (presence of fungal broad, non-septate hyphae in pathologic examination or growth of Mucormycosis on sabouraud dextrose agar (SDA) and indirect mycologic test (fungal PCR) in tissue biopsy

^2^Noxafil 40 mg/ml oral suspension; Manufacture by MSD company

### Laboratory, pathology, immunologic and imaging workup

**Fig. 1:**
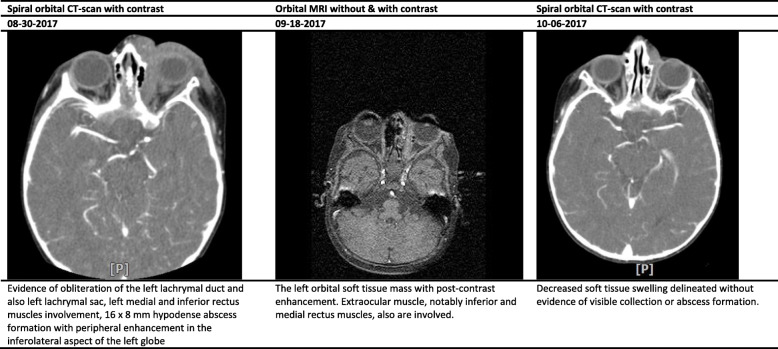
Summary findings of orbital CT-scan and MRI images at admission and during treatment in case 1

**Fig. 2:**
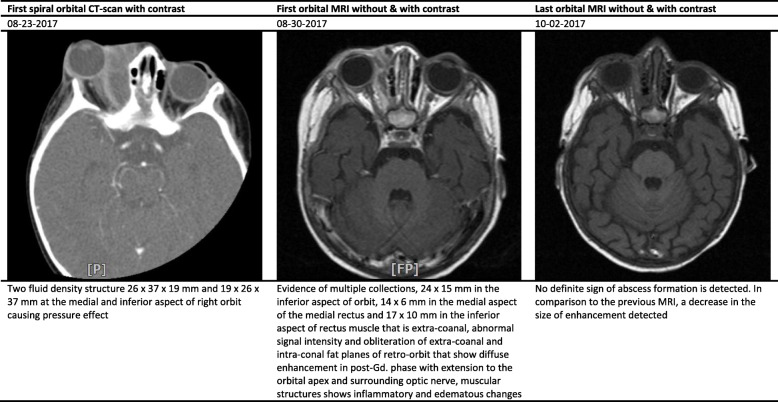
Summary findings of orbital CT-scan and MRI images at admission and during treatment in case 2

**Fig. 3:**
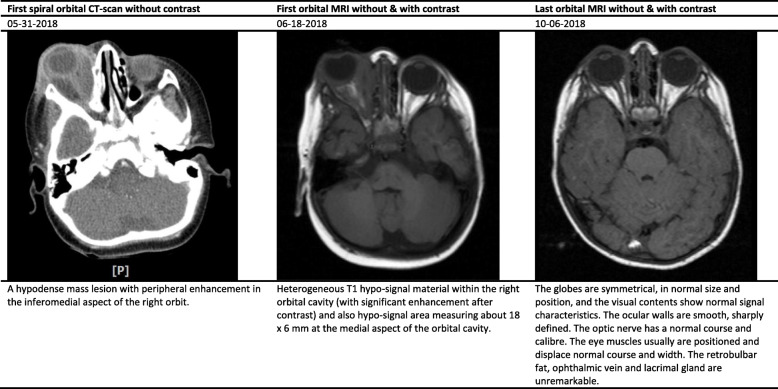
Summary findings of orbital CT-scan and MRI images at admission and during treatment in case 3

**Fig. 4:**
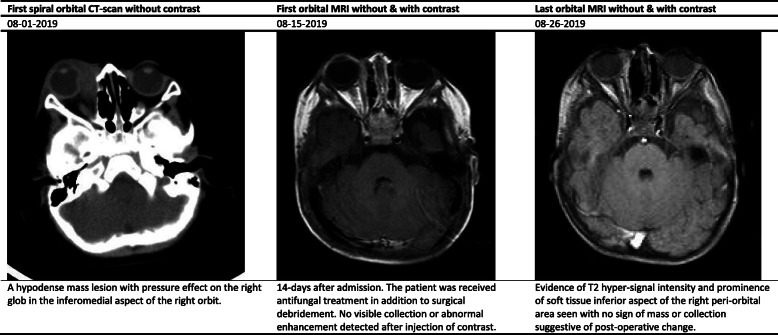
Summary findings of orbital CT-scan and MRI images at admission and during treatment in case 4

**Fig. 5:**
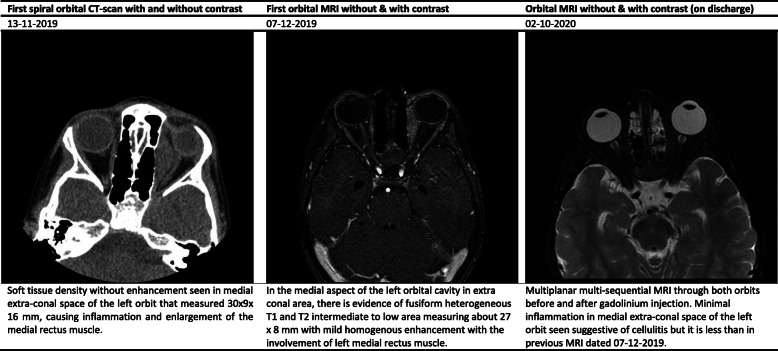
Summary findings of orbital CT-scan and MRI images at admission and during treatment in case 5

**Fig. 6:**
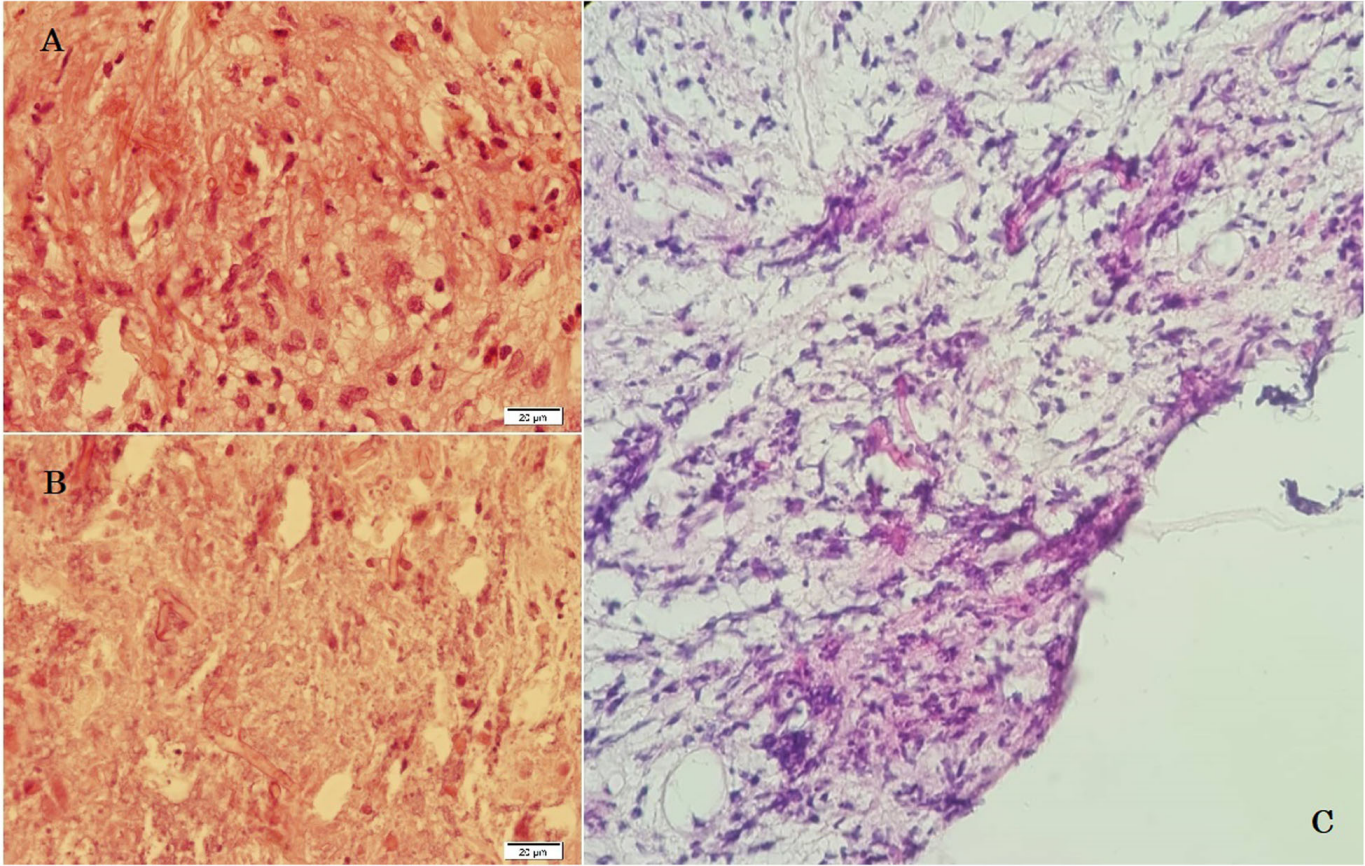
Non-septate broad hyphae in the necrotic background with acute inflammatory cell infiltration, H&E stain (× 400). **a, b,** and **c** represent a microscopic examination of orbital masses in cases 1, 3, and 4, respectively

The white blood cell (WBC) count, hemoglobin (Hb), and platelet count ranged between 9.7–33.1 (cell/mm^3^), 9.9–12.7 (mg/dl), and 383–640 (thousand/mm^3^), respectively (Table 2). Interestingly, the erythrocyte sedimentation rate (ESR) and C-reactive protein (CRP) values significantly increased in studied cases. All cases underwent specific diagnostic investigation, including molecular tests as we described previously [12], and immunologic workup (Table 2). All patients regularly assessed by orbital/PNS CT-scan and MRI (with and without contrast) initially and serially after surgical intervention until full imaging recovery. Results of serial orbital MRIs have summarized in Figs. 1, 2, 3, 4 and 5. The histopathological examination of the orbital biopsies revealed fungal elements by Hematoxylin and Eosin (H&E) staining in all studied patients (Fig. 6).

**Table 2:** Laboratory findings of five cases with orbital mucormycosis

	Case 1	Case 2	Case 3	Case 4	Case 5
WBC (X10^3^)	9.7	12.2	33.1	10.8	17.3
Hb (mg/dl)	12.7	10.2	11.3	9.9	10.9
Plt (X10^3^)	392	476	640	500	383
BUN/Cr (mg/dl)	6/0.4	5/0.4	12/0.5	6/0.4	15/0.5
Na (mg/dl)	136	141	141	138	142
K (mg/dl)	4.5	5	4.6	4.6	5.3
ESR	41	21	31	22	64
CRP	18	7	> 150	4	1
Serum immunoglobulin levels^1^	Nl^a^	Nl^a^	Nl^a^	Nl^a^	Nl^a^
Dihydrorhodamine (DHR) test	93	130	167	117	90
Peripheral blood flow cytometry^2^					
CD3	68%	56%	86%	72%	59%
CD4	37%	33%	55%	39%	37%
CD8	30%	21%	28%	25%	14%
CD16	10%	5%	5%	4%	13%
CD19	22%	42%	15%	23%	28%
CD20	22%	39%	15%	23%	28%
CD56	10%	5%	5%	4%	13%
Interferon Gamma receptor	Normal	Normal	Normal	Normal	Normal
KOH	Non-septate broad hyphae	Non-septate broad hyphae	Non-septate broad hyphae	Non-septate broad hyphae	Non-septate broad hyphae
Culture^3^	Negative	Positive	Positive	Negative	Negative
Mucormycosis PCR^4^	Positive	Negative	Negative	Positive	Positive
Microscopic examination by (H&E) staining	Positive	Positive	Positive	Positive	Positive

*WBC* White blood cell count, *Hb* Hemoglobin, *Plt* platelet count, *BUN/Cr* blood urea nitrogen/creatinine, *Na* serum sodium, *K* serum potassium, *ESR* Erythrocyte sedimentation rate, *CRP* C-reactive protein, *H&E* Hematoxylin and Eosin (H&E) staining

^1^Including serum IgG, IgM, IgA, and IgE levels

^2^Immunophenotyping of the patient’s peripheral blood mononuclear cells (PBMCs) Including CD3, CD4, CD8, CD16, CD19, CD20, and CD56

^3^All isolates were cultured on Sabouraud dextrose agar

^4^Nested PCR was performed with set of primer for *Mucormycosis* (as authors described previously reference [12])

^a^Within normal limits for age

## Discussion and conclusion

Herein, we report orbital mucormycosis in five immunocompetent children without underling disease or known immunodeficiency successfully managed with medical and surgical interventions. Although invasive forms of mucormycosis have been described and well-known in immunocompromised hosts, such as bone marrow transplantation (BMT), aggressive immunosuppressive therapy in hematological malignancies and diabetic patients [3, 13–15], it is scarce in healthy individuals [2, 3, 8–11, 16]. Based on a retrospective global analysis of 101 cases of mucormycosis in France reported by Lanternier et al., all except one have underlying risk factors [17]. Interestingly, the median number of underlying risk factors per patient was 2, while 29% of them harbouring ≥three risk factors, including hematological malignancy, HSCT, graft versus host disease (GVHD), diabetes mellitus, corticosteroids, neutropenia, and solid organ transplantation. Eight of 101 cases reported in children with a median age of 13.4 years. The identified underlying conditions were trauma (50%) and hematological malignancy (50%), which mainly localized on skin. Sino-orbital and isolated ocular mucormycosis reported in five and one cases, respectively [17].

In another large case series reported by Maureen et al. in 929 cases of mucormycosis, 19% were immunocompetent without known underlying conditions at the time of infection, penetrating trauma, surgery, and burns were the leading causes. The most localization sites were sinus (39%; including 8% Sino-orbital), pulmonary (24%), cutaneous (19%), cerebral (9%), gastrointestinal (7%), and kidney (2%). None of the reported cases was isolated ocular mucormycosis [18].

While pulmonary and isolated sinusal, rhino-cerebral, rhino-sinuso-orbital, or rhino-orbito-cerebral invasive forms are the most common clinical presentation in immunocompromised patients [1]; invasive mucormycosis usually present as skin and soft tissue infections (SSTIs) in immunocompetent individuals following burn and trauma [19]. However, the site and severity of disease are widely varied based on predisposing factors [2, 3]. Rhizopus oryzae has reported as the most common etiologic cause of rhino-sinuso-orbital mucormycosis [20].

Commonly identified risk factors for orbital mucormycosis consist of hematological malignances (17 to 77% of cases) [15, 20], burn [21], placement of the prosthetic device, [22], post-traumatic (after dental extraction) [23, 24] and diabetes mellitus [2, 3, 25].

Isolated orbital mucormycosis rarely reported in immunocompetent individuals without well-known predisposing factors [16, 26–29] and usually including rhino-orbital, orbito-cerebral, facio-orbital, or other non-isolated forms [30–32].

Early diagnosis is essential to reduce severe morbidity (for example, eye exenteration) and also mortality [6, 11]. Cerebral involvement (CNS seeding) [18, 33] and other rare complications, such as orbital apex syndrome [34, 35], also reported in immunocompetent patients.

The standard approach for the treatment of mucormycosis usually based on the combination of liposomal amphotericin B, timely surgical debridement of involved tissues (especially in sinu-orbital and cutaneous forms), and finally control of underlying conditions [19, 36, 37]. Liposomal formulation of amphotericin is preferred, when available, to reduce the risk of infusion-related reaction and nephrotoxicity [38]. Repeated surgical debridement of necrotic tissue is warranted when remaining involved tissue start to progressed despite adequate initial aggressive debridement.

Although the mortality is very high in immunocompromised patients, the prognosis seemed promising when effective systemic antifungals, early surgical drainage of pus, and orbital decompression considered in immunocompetent children [6, 37]. Delayed effective antifungal therapy (more than 6 days after diagnosis) could result in a 2-fold increase in mortality rate, as studied by Chamilos et al., in 70 patients with hematologic malignancy [6]. In contrast to other reports, none of the patients undergone aggressive surgical debridement, including exenteration surgery.

The duration of treatment may be related to several factors. An underlying condition, time from illness onset to hospital admission, route of infection, distant seeding (cerebral involvement), age, an early response to treatment, timely surgical intervention, and need for repeated debridement, all could affect the duration of treatment and outcome [18].

As highlighted in Table 1 orbital mucormycosis which presenting with septal cellulitis is quite rare in healthy children, so any information regarding the patient’s background, predisposing factors, and clinical features of orbital mucormycosis are helpful to guide the physician to suspect invasive mucormycosis in any child with pre-septal/septal cellulitis.

The age of infected children is an important finding in our study. The mean age (±Std. Deviation) of studied cases was 16 (±11.94) months, with a median of 10 months (range between 7 to 36 months).

History of gardening and contact with dust found in two cases. History of iatrogenic trauma (probing) detected in one case. The delay time interval from illness onset to hospital admission is another important finding in our patients. The mean delay time (±SD) was 3.6 (±2.5) weeks, with a median of 4 weeks (range between 1 to 6 weeks).

In comparison with bacterial preseptal/septal cellulitis, on admission, laboratory tests revealed comparable ESR but higher CRP levels [39, 40]. The mean ESR/CRP (±SD) was 35.8/36 (±17.7/64), with a median 31/7. The mean WBC count (±SD) was 16,600 (±9600), with a median of 12,200 (range between 9700 to 33,100), which also was slightly higher than bacterial preseptal/septal cellulitis [39, 40].

Diagnostic orbital CT-scan is another useful and informative modality in the timely diagnosis of orbital mucormycosis. Some experts recommend orbital CT for any children with possible preseptal cellulitis to find other differential diagnoses such as subperiosteal abscess that presented similar to preseptal cellulitis without classic signs and symptoms, including proptosis, visual decrease, or ophthalmoplegia [41]. Based on our experience orbital CT-scan should be advocated in any case of preseptal cellulitis who has marked lid inflammation with edema extending beyond the lid margins, high white blood count elevated CRP levels, and previous antibiotic therapy.

Despite discussion mentioned above, orbital mucormycosis could be developed in the absence of any apparent risk factors (as noted in case 3 and 4), and attention should be paid to the history of gardening, and minor trauma to the lachrymal duct (probing), and exposure to corn and fodder storage and hay in young children.

Finally, to obtain a successful outcome, frequent debridement has a critical role in the management of such patients. To guide timely surgical intervention, serial orbital/PNS CT/MRI, with and without contrast, is mandatory.

In conclusion, these unique cases emphasize the need for a high index of suspicion for the unusual cause of orbital infections, especially fungal infections, in otherwise healthy patients with no apparent predisposing factors. Although mucormycosis predominantly reported in patients with hematological malignancies, it should be considered as part of the initial workup in each patient diagnosed with preseptal/septal cellulitis unresponsive to empirical broad-spectrum antibiotic therapy, high CRP levels, and severe proptosis.

## Data Availability

All data generated or analyzed during this study included in this article.

## Abbreviations

MG: Marcus Gunn
VA: Visual acuity
EOM: Extra-ocular muscle
CT: Computed tomography
PCR: Polymerase chain reaction
FESS: Functional endoscopic sinus surgery
NLDO: Nasolacrimal duct obstruction
PNS: Paranasal sinus
MRI: Magnetic resonance image
ER: Emergency room
WBC: White blood cell
Hb: Hemoglobin
ESR: Erythrocyte sedimentation rate
CRP: C-reactive protein
DHR: Dihydrorhodamine test
BMT: Bone marrow transplantation
GVHD: Graft-versus-host disease
SSTI: Skin and soft tissue infections
CNS: Central nervous system
